# Negative representations of night-shift work and mental health of public hospital healthcare workers in the COVID-19 era (Aladdin survey)

**DOI:** 10.1186/s12913-023-09101-7

**Published:** 2023-02-22

**Authors:** Lorraine Cousin Cabrolier, Vincent Di Beo, Fabienne Marcellin, Olivia Rousset Torrente, Véronique Mahe, José Maria Valderas, Olivier Chassany, Patrizia Maria Carrieri, Martin Duracinsky

**Affiliations:** 1 Unité de Recherche Clinique en Economie de La Santé (URC-ECO), AP-HP, Hôpital Hôtel-Dieu, 75004 Paris, France; 2 Université Paris Cité, ECEVE UMR 1123, Inserm, Faculté de Médecine, Paris, France, Paris, 75010 France; 3 Aix Marseille Univ, Inserm, IRD, SESSTIM, Sciences Economiques & Sociales de La Santé & Traitement de L’Information Médicale, ISSPAM, Marseille, France; 4 Service de Santé Au Travail, Hôpitaux Lariboisière-Fernand Widal, AP-HP Nord, Paris, France; 5 Department of Medicine, NUS Yong Loo Lin School of Medicine, National University of Singapore, Singapore, Singapore; 6 Département de Médecine Interne Et d’immunologie Clinique, Hôpital Bicêtre, AP-HP, 94275 Kremlin Bicêtre, France

**Keywords:** Occupational health, Shift work, Health workers, Mental health

## Abstract

**Background:**

Many risk factors impact the health of hospital night workers, which can lead to physical and mental health disorders. During the recent period, night hospital workers have been particularly stressed. This study therefore aims to: (i) To document the prevalence of depression, anxiety, sleep disorders, and symptoms suggestive of post-traumatic stress disorder in night shift workers (NSHW) working in Parisian public hospitals after France’s first COVID-19 wave ended; (ii) To estimate the effect of negative representations and perceptions of night shift work on these mental health outcomes.

**Methods:**

An observational cross-sectional online survey of NSHW (June to September 2020) in 39 public hospitals in Paris, France. Standard scales were used to measure mental health outcomes. Weighted multinomial logistic regression models supported the identification of predictors of depression (score > 10 on the Hospital Anxiety and Depression Scale, HADS, for depression), anxiety (score > 10 on the HADS for anxiety), severe insomnia (score > 21 on the Insomnia Severity Index, ISI) and symptoms suggestive of post-traumatic stress disorder (score > 36 on the Impact of Event Scale-Revised, IES-R).

**Results:**

The weighted prevalence rates [95% confidence interval] of depression, anxiety, severe insomnia, and symptoms of post-traumatic stress disorder were, respectively, 18.9% [16.5–21.2], 7.6% [6.0–9.1], 8.6% [6.9–10.2] and 11.7% [9.7–13.6]. After multiple adjustment, organizational changes in NSHW professional lives due to the COVID-19 pandemic (such as moving to another hospital department and modified schedules) and NSHW-perceived negative representations of night work were significantly associated with all studied mental health outcomes.

**Conclusion:**

Our findings confirm the importance of monitoring mental health and sleep quality among NSHW in Parisian public hospitals, even more during health crises. Multilevel interventions aiming at reducing negative representations and improving work organization are urgently needed to improve overall health of this frontline healthcare providers group.

**Supplementary Information:**

The online version contains supplementary material available at 10.1186/s12913-023-09101-7.

## Background

The organization of healthcare, including work shifts, staff density and contact with the public, can put healthcare professionals at risk of impaired mental health [1]. For example, doctors and nurses experience high levels of stress and anxiety due, among other factors, to their professional duties [2]. Even before the pandemic, hospital workers were subjected to a high level of violence at work [3] and a high risk of burnout in several countries [4–6]. Night-shift healthcare workers (NSHW), like other healthcare professionals, need excellent mental health to effectively and efficiently perform their jobs. However, they may be at particular risk of mental health impairment [7], as they are exposed to alterations of circadian rhythms [8, 9] and possible sleep disorders [10], two known determinants of mental health problems [11]. Furthermore, previous studies highlighted that night-shift workers had significant levels of stress and depressive symptoms [12], and this may also be the case for NSHW. While physical health issues related to sleep and nutrition have been studied in this population, mental health in NSHW is a mostly underexplored dimension.

Some studies have identified negative representations or stigmatization of night work and night-shift workers by day workers, relatives and patients, who associated night work with “rest time”, “latency” and “emptiness”, [13–15]. These representations may negatively impact the mental health of NSHW. To date, very little research has been conducted on this issue.

The ongoing COVID-19 pandemic has led to massive disruption in hospital organization and patient care [16, 17], causing great stress and anxiety in the general population [18, 19], but also in frontline health professionals [20, 21]. During the first wave of COVID-19 in France in March–May 2020, the geographical region served by the 39 structures in the Public Assistance of Paris Hospital (AP-HP) hospital network was among the three regions in France most affected in terms of numbers of infections and hospital saturation. The network’s 100,000 strong workforce—12,000 of whom are NSHW—faced substantial changes in terms of working environment and work organization; these changes included prolonged working hours, transfer to COVID-19 specific services, and having to switch between different hospital departments often at short notice [16].

The main objective of the present study was to document and estimate the prevalence of depression and anxiety, sleep disorders and symptoms suggestive of post-traumatic stress disorder in this population, as well as the prevalence of the three elements of NSHW-perceived negative representations listed above (i-iii). i) perceived negative representations of night work by day workers, patients, and relatives, ii) self-negative representations of night work, and iii) COVID-19-related changes to the working environment and work organization, influenced AP-HP NSHW mental health outcomes (depression, anxiety, post-traumatic stress disorder) and quality of sleep.

## Methods

### Design and settings

Aladdin is a cross-sectional study of NSHW working in all 39 structures of the AP-HP public hospital network which covers Paris and the surrounding area. It aimed to portray the situation of these workers during the COVID-19 pandemic by collecting sociodemographic and economic data, as well as data on work-related characteristics, perceived health, quality of working life, mental health, use of psychoactive substances and sleep quality [22]. The study questionnaire was created using NetSurvey ® and was made available on personal and professional devices between 15 June and 15 September 2020 [23]. The Aladdin instrument has been tested for understanding through a cognitive debriefing [24] on a sample of NSHWs before the start of the survey. Several strategies were used to disseminate the study. The link to the questionnaire was sent to healthcare professionals’ email addresses, posters were pinned up in hospital wards, medical students distributed flyers at night, and flyers and posters were also sent to NSHW team managers. Reminders were sent every week and then every month to maximize participation.

### Participants

Participants in Aladdin included health managers, senior managers, nurses, assistant nurses, lab technicians, midwives, childcare assistants, X-ray technicians, and administrative professionals who worked at night. Some participants were newly assigned to night-shift work. In order to keep the study population homogeneous, physicians were excluded from the analyses as they constitute a subgroup with specific characteristics. Physicians in the AP-HP network primarily work during the day. When working nights, they work on an on-call basis only. Accordingly, the amount of night work they perform is lower and less regular than that of other NSHW. Night work was defined as working between 9:00 p.m. and 6:00 a.m. a minimum of twice a week.

### Ethics

Participants gave informed consent to participate in Aladdin by checking a box after reading the information note. Aladdin was approved in March 2020 by an independent Ethics Committee (Lyon 2, ID RCB202-A00495-34).

### Instruments and study variables

#### Assessment of mental health and sleep disorders

The Hospital Anxiety and Depression Scale (HADS) [25] was used to assess depression and anxiety. Its scores range from 0 to 21 for each of the two conditions, with scores > 7 and ≤ 10 indicating a probable case, and > 10 indicating a definite case. The respective sensitivity and specificity are respectively 88% and 69% for the cut-off point of 7, and 74% and 83% for the cut-off point of 10 [26].

To assess insomnia, we used the French validated version [27] of the Insomnia Severity Index (ISI).The ISI score ranges from 0 to 28; a score between 15 and 21 indicates moderate insomnia, while over 21 indicates severe insomnia. With these cut-off points, the sensitivity is 80% and the specificity 72% [28].

We assessed post-traumatic stress disorder using the French validated version of the Impact of Event Scale-Revised (IES-R) [29]. The IES-R score ranges between 0 and 88; scores over 23 indicate a mild to moderate psychological impact, while scores over 36 indicate a severe psychological impact and symptoms of post-traumatic distress. The sensitivity of the scale is 1.0 and the specificity is 0.78 [30].

These scales were chosen because of their regular use in studies and surveys in specific and general populations as well as in routine patient management. Choosing these scales allowed us to compare the results with those of other studies and populations.

#### Assessment of NSHW-perceived negative representations of their work

We defined the negative representations process as a process close to the stigmatization process. It is the labeling, stereotyping, separation, status loss, and discrimination of a person with specific characteristics [31]. Stigma and negative representations can be broken down into different forms [32]: public stigma, self-stigma, perceived stigma, label avoidance, stigma by association, structural stigma and health practitioner stigma. In our study, we explored NSHW-perceived negative representations and self-negative representations using questions created and cognitively tested by the Aladdin research team for a previous study [33].

Perceived negative representations can be defined as an individual’s belief that others have a negative cognition and stereotype about him/her [34]. We measured perceived negative representations using eight questions adapted from different stigma scales [35–37]. These questions focused on: 1) underestimation of NSHW work by day colleagues, by close relatives, by partners, and by patients; 2) negative feedback of their work by day workers; 3) isolation from the rest of the work team; 4) ability to valorize their work to others. The respondent rated the occurrence of all the elements listed in these four focus areas with options ranging from never to always.

Self-negative representations are defined as the internalization of stereotypes and prejudices [38]. We measured it using questions which focused on: 1) the extent to which NSHW agreed that night-time working duties were less important than day-time duties in terms of patient care; 2) the extent to which NSHW agreed that the night-time workload was smaller than the day-time workload.

#### Assessment of COVID- 19 pandemic-related organizational changes

The dimensions explored in the questionnaire’s COVID-19-related items were: 1) changes in work organization (place of work, schedule, rhythm, activity); 2) management of the COVID-19 crisis at work; 3) feelings related to COVID-19, specifically NSHW fear of and perceived vulnerability to contracting COVID-19 and their trust in the government’s decisions and protective measures; 4) use of psychological support to help manage the consequences of the pandemic on their mental health.

### Statistical methods

Data were weighted using the raking ratio method [39] in order to be representative of the entire night staff of the AP-HP network in terms of sex, age (using 5-year age classes), and professional category. Five professional categories were created as follows: nurses (both specialized and non-specialized), assistant nurses and laboratory technicians, managers, midwives and other. All analyses were performed on weighted data.

Descriptive statistics were used to document the characteristics of the study population and to estimate the prevalence and associated 95% confidence interval (CI) of definite cases of depression and anxiety (HADS), sleep disorders (ISI) and symptoms suggestive of post-traumatic stress disorder (IES-R) in the whole study population. We then performed comparisons between the five different professional categories using a Chi-Squared and Wald test for categorical and continuous variables, respectively.

Weighted multinomial logistic regression models were used to identify correlates of the four mental health outcomes defined above. First, we performed univariable logistic regression models with the following variables: socio-demographic characteristics (sex, age, and perceived financial status), work-related characteristics (profession, position, seniority, working hours, travel time to work), and health-related characteristics (physical activity, history of psychiatric disorders, history of harassment at work, history of COVID-19 infection). Second, all variables with a liberal *p*-value smaller than 0.200 in the univariable analyses were entered in the multivariable analysis. Third, a manual backward selection procedure was used to build the final multivariable model, which included only significant variables (*p* < 0.050). We used Stata version 14.2 for Windows software (StataCorp, College Station, Texas, USA) for all statistical analyses.

## Results

### Participants

Of the 12,000 NSHW in the AP-HP network, 1,585 (13.2%) answered the Aladdin study questionnaire. Of these, 1,200 participants had complete data for mental health outcomes (HADS, IES-R and ISI scales) and constituted the study sample for the present analysis.

Mean age of the study sample was 39.4 years (standard deviation: 11.8 years), 78.2% [95% CI = 75.7–80.8] were women and 50.7% [47.7–53.6] had children. Nurses represented more than half (53.6%) of the sample. The characteristics of the study sample are described in Table 1 and Additional file 1: Appendix 1.

**Table 1:** Characteristics of the study sample and multinomial regression models of factors associated with anxiety, depression, severe insomnia and symptoms suggestive of post-traumatic stress disorder (Aladdin survey, *n* = 1,200 NSHW with available data on mental health outcomes). Results on caseness or each model. The table with the complete models (including non-significant variables) is presented in Additional file 1: Appendix 1

		**HADS Anxiety** **( ref: absence of symptomatology)**		**HADS depression** **( ref: absence of symptomatology)**		**Index of insomnia severity** **( ref: Absence or Sub-threshold)**		**Post-traumatic stress disorder** **( ref: No particular stress)**	
	**Whole study sample**	**Tested: caseness**		**Tested: caseness**		**Tested: severe insomnia**		**Tested: post-traumatic stress**	
**Characteristics**	**% of NSHW or mean (SD)**	**aRRR**^**1**^** [95% CI]**	***p*-value**	**aRRR**^**1**^** [95% CI]**	***p*-value**	**aRRR**^**1**^** [95% CI]**	***p*-value**	**aRRR**^**1**^** [95% CI]**	***p*-value**
**Female gender **^**a**^	78.3	**1.98 [1.26–3.11]**	0.003						
**Age** – per each year increase	39.4 (11.8)	**0.97 [0.95–1.00]**	0.025			**0.94 [0.92–0.97]**	< 0.001		
**Perceived financial status**									
-Financially comfortable/ gets by	40.3	ref		ref		ref		ref	
-Has to be careful	46.4	**1.65 [1.12–2.42]**	0.011	**1.83 [1.05–3.20]**	0.033	**2.20 [1.29–3.77]**	0.004	**2.48 [1.54–3.99]**	< 0.001
-Financial difficulties	13.3	**3.46 [2.00–5.98]**	< 0.001	**3.15 [1.52–6.54]**	0.002	**4.68 [2.31–9.47]**	< 0.001	**4.33 [2.38–7.85]**	< 0.001
**Work-related characteristics**									
**Professional category**									
-Nurses (specialized or not)	53.6	ref							
-Assistant nurses/technicians	36.9	0.85 [0.57–1.26]	0.423						
-Midwives	4.1	0.45 [0.17–1.16]	0.096						
-Managers	0.8	2.54 [0.42–15.49]	0.312						
-Other	4.6	**2.51 [1.07–5.87]**	0.034						
**Seniority as a night-shift worker** – per year increase	9.2 (8.5)							**0.96 [0.94–0.99]**	0.009
**Health-related characteristics**									
**Physical activity **^**b**^	54.2			**0.55 [0.34–0.89]**	0.015	**0.62 [0.39–0.98]**	0.041		
**History of psychiatric disorders (depression, bipolar disorder, etc.) **^**b**^	5.1	**4.19 [2.10–8.35]**	< 0.001			**2.54 [1.06–6.08]**	0.036		
**History of harassment at work **^**b**^	21.0	**1.62 [1.10–2.40]**	0.016			**1.82 [1.06–3.12]**	0.031	**2.85 [1.87–4.36]**	< 0.001
**History of SARS-CoV-2 infection **^**b**^	18.5					**2.02 [1.10–3.69]**	0.022		
**Work-related perceptions**									
**Night-shift work is underestimated by colleagues working on day **^**d**^									
Always, regularly	65.0	0.84 [0.57–1.24]	0.379	**2.10 [1.16–3.82]**	0.014				
**Night-shift work is underestimated by patients **^**d**^									
Always, regularly	17.7	**1.86 [1.23–2.79]**	0.003	**2.02 [1.21–3.39]**	0.008	**1.81 [1.05–3.12]**	0.031		
**Day duties are more important than night duties in terms of patient care**^**c**^									
Strongly agree, agree	23.5	**1.50 [1.02–2.20]**	0.038	**2.25 [1.36–3.74]**	0.002	**2.27 [1.38–3.75]**	0.001	**1.96 [1.26–3.04]**	0.002
**Work organization: changes since the beginning of the COVID-19 pandemic**									
**Switch to night-shift work**	4.8			**3.22 [1.37–7.57]**	0.007				
**Change of activity to manage COVID-19 patients **^**b**^	18.6	**1.54 [1.03–2.31]**	0.035			**1.81 [1.07–3.05]**	0.027	**2.07 [1.30–3.28]**	0.002
**Feelings related to the COVID- 19 pandemic**									
**Satisfied with the information on COVID-19 provided by employer **^**b**^	31.5	**0.48 [0.32–0.73]**	0.001						
**Afraid of contracting COVID-19 at work **^**b**^	65.6	**1.75 [1.19–2.56]**	0.004					**2.12 [1.31–3.44]**	0.002
**Considered protective measures to be inadequate **^**c**^									
- strongly agree, agree	27.4					**1.90 [1.18–3.06]**	0.008	**1.62 [1.06–2.48]**	0.027

*CI* Confidence interval, *NSHW* Night-shift healthcare worker, *SD* Standard deviation

^1^*aRRR* Adjusted Relative Risk Ratio

^**a**^Response “Male” set as reference

^b^Response “No” set as reference (modality Yes is tested)

^**c**^Responses “strongly disagree”, “disagree” or “indifferent” were combined and set as reference

^**d**^Responses “Never”, “rarely”, or “sometimes” were combined and set as reference

### Description of participants’ mental health

**Fig. 1:**
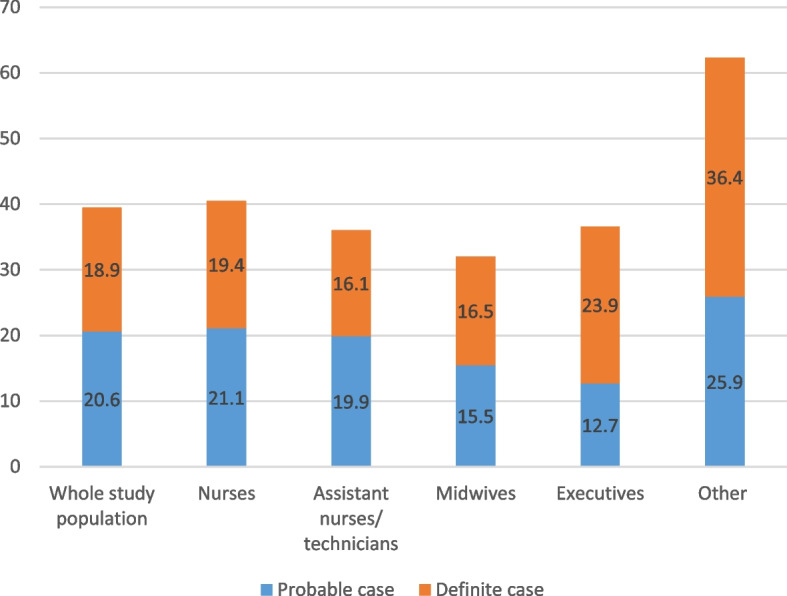
Prevalence of depression (HADS) according to professional category

**Fig. 2:**
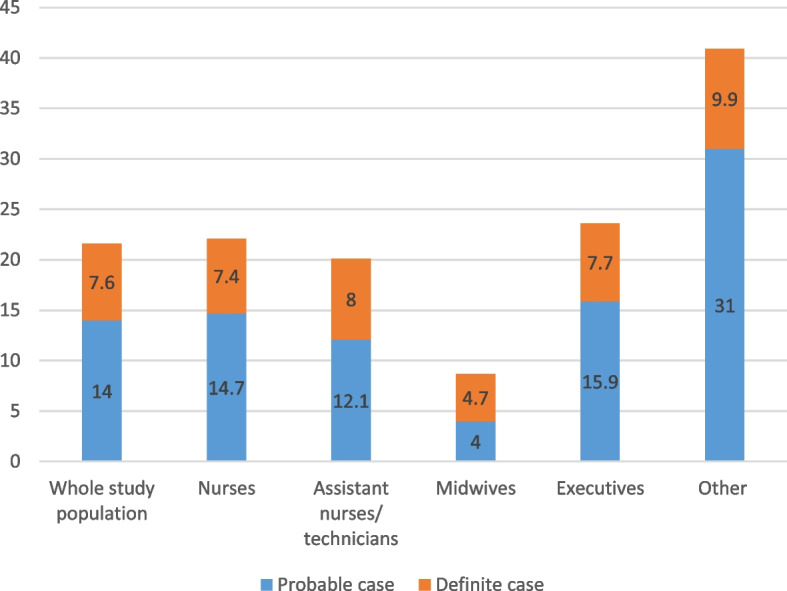
Prevalence of anxiety (HADS) according to professional category

**Fig. 3:**
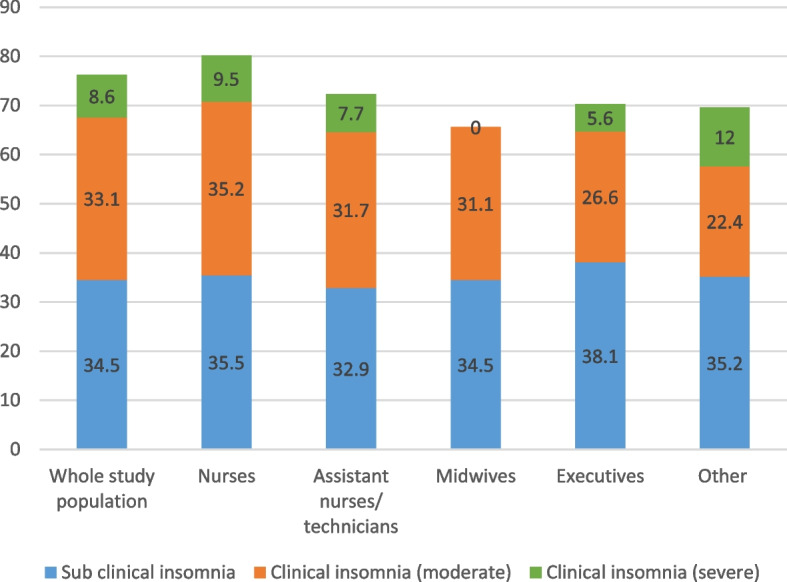
Prevalence of severe clinical insomnia (ISI) according to professional category

**Fig. 4:**
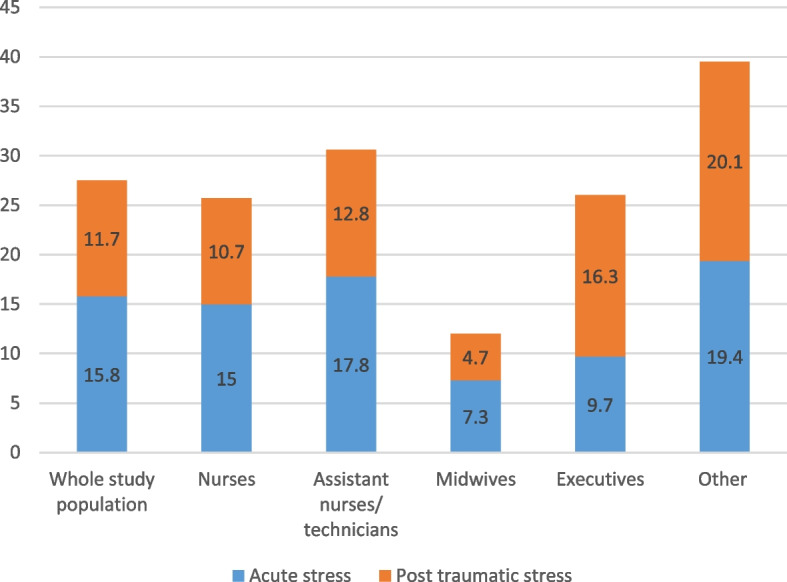
Prevalence of symptoms suggestive of post traumatic stress disorders (IES-R) according to professional category

In our sample, 23.0% of respondents had at least one of the four severe mental health issues. The prevalence rates [95% CI] of definite cases of anxiety and definite cases of depression were 18.9% [16.5–21.2] and 7.6% [6.0–9.1], respectively (Figs. 1 and 2). They differed significantly between professional categories (Wald test, *p* = 0.011; Figs. 1 and 2). The professional category ‘other’ was the most likely to have definite cases of both conditions, followed by ‘managers’ (Figs. 1 and 2). Only a quarter of respondents (24.0% [21.4–26.5] and 8.6% [6.9–10.2]) did not suffer from sleep disorders were defined as having severe insomnia (Fig. 3). Symptoms of post-traumatic stress disorder prevalence was 11.7% [9.7–13.6] (Fig. 4). More information about the mental health of participants according to their professional category is reported in Additional file 1: Appendix 1.

### Negative representations of night shift work and isolation

We estimated two dimensions of the negative representations of night work: perceived and self-negative representations. A majority of NSHW (65.0% [62.1–67.8]) felt that their day colleagues underestimated their work. A minority felt that their relatives and patients underestimated their work (21.0% [18.6–23.4] and 17.7% [15.5–19.9], respectively). With regard to isolation from the rest of the team, a large majority did not attend staff meetings or department/clinical meetings (86.3% [84.3–88.4] and 95.2% [93.8–96.6], respectively). One fifth (20.7% [18.4–23]) reported they felt isolated in their work. Just under half (46.6% [43.7–49.6]) reported they felt empowered and were capable of valorizing their job. In terms of the perception that day-time work activities are more important than night-time activities for patient care, 23.5% [21.0–26.0] of NSHW agreed (responses: ‘I agree’ / ‘I totally agree’) with this assertion, and 39.1% [36.2–41.9] agreed that the day-time workload was greater than the night-time workload.

### COVID-19-related organizational changes

We investigated changes to work organization since the beginning of the COVID-19 pandemic. Sixty-three percent of respondents reported at least one change to adapt to the health crisis. More specifically, 36.8% [33.9–39.6] reported an increase in working hours, 25.1% [22.6–27.6] changed hospital department, and 30.2% [27.5–32.9] moved to another unit in the same department. Overall, 18.6% [16.4–20.8] of respondents changed their daily work activities to manage COVID-19 patients.

### Perception of factors related to COVID-19

A majority of respondents (77.7% [75.2–80.2]) felt more vulnerable to COVID-19 infection because of their professional activity, 65.6% [62.8–68.5] reported being afraid of contracting COVID-19, and 90.8% [89.1–92.6] reported they feared transmitting it to their close relatives. In addition, 59.9% [56.9–62.8] reported finding it difficult to implement protective measures against COVID-19 at work while 27.4% [24.8–30.1] considered these measures inadequate. More than half (58.5% [55.6–61.4]) considered that they received insufficient or incomplete information from their employer about the COVID-19 pandemic.

### Multivariable logistic regressions

The impact of NSHW-perceived negative representations of their work and the impact of organizational changes caused by the COVID-19 pandemic on mental health were identified in multivariable regressions. The results of the multivariable logistic regressions, using adjusted relative risk ratios (aRRR) and their 95% CI, are shown in Table 1, and the full final models (with the addition of aRRR associated to probable case of anxiety/depression, sub clinical or clinical insomnia and acute stress) are described in Additional file 1: Appendix 2.

With regard to changes in professional life due to COVID-19 pandemic, switching to night-shift work was significantly associated with definite depression (aRRR [95% CI] = 3.22 [1.37–7.57]). Change in work activity to manage COVID-19 patients was significantly associated with definite anxiety (1.54 [1.03–2.31]), with severe insomnia (1.81 [1.07–3.05]) and with symptoms suggestive of post-traumatic stress disorder (2.07 [1.30–3.28]).

With regard to feelings related to COVID-19, being satisfied with the information on the disease received from their employer was associated with a lower risk of definite anxiety (0.48 [0.32–0.73]) (Additional file 1: Appendix 2).

By contrast, the fear of contracting COVID-19 at work was associated with a higher risk of both definite anxiety (1.75 [1.19–2.56]) and symptoms suggestive of post-traumatic stress disorder (2.12 [1.31–3.44]). Similarly, considering that recommended COVID-19 protective measures were inadequate was associated with severe insomnia (1.90 [1.18–3.06]) and post-traumatic stress disorder (1.62 [1.06–2.48]).

NSHW-perceived negative representations of their work was associated with poorer mental health. Specifically, those who reported that day colleagues underestimated their work were more likely to definitely have depression (2.10 [1.16–3.82]), while those who perceived that patients underestimated night work were more likely to definitely have anxiety (1.86 [1.23–2.79]), depression (2.02 [1.21–3.39]), and severe insomnia (1.81 [1.05–3.12]). NSHW self-negative representations was also associated with impaired mental health. Specifically, agreeing that day-time working duties are more important than night-time duties in terms of patient care was associated with definitely having anxiety (1.50 [1.02–2.20]), definitely having depression (2.25 [1.36–3.74]), severe insomnia (2.27 [1.38–3.75]), and symptoms of post-traumatic stress disorder (1.96 [1.26–3.04]). All these associations are summarized in Table 1. Logistic regressions performed only on the nurses group showed stability of results between the full sample and the sample restricted to nurses only.

## Discussion

Using data from the Aladdin survey, the present study documented the mental health of NSHW in the AP-HP public hospital network during the first wave of the COVID-19 pandemic in France, and explored the potential impact of two factors on mental health, namely perceived negative representations of night-work, and COVID-19-related organizational changes. In our weighted sample, which was representative of the 12,000 NSHW in the network, in terms of sex, age, and professional category, 23% of respondents had at least one of the four severe mental health issues explored. Specifically, twenty percent of participants probably or definitely had depression, while 40% probably or definitely had anxiety. Seventy-five percent of respondents suffered from sleep disorders (light, moderate or severe), and almost 12% suffered from symptoms of post-traumatic distress. Finally, mental health varied significantly according to professional category.

### Comparison with the French general population’s mental health

**Fig. 5:**
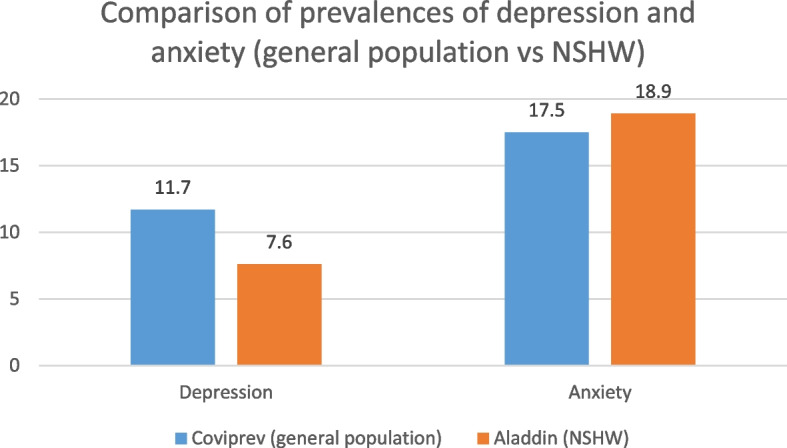
Comparison of the prevalences of depression and anxiety (general population vs NSHW)

During the first wave of the COVID-19 pandemic, three different studies (COVIPREV [40], COCLICO [41] and EPICOV [42]) investigated factors impacting mental health in the French general population. While the HADS was used in COVIPREV (as in Aladdin), the scales used in COCLICO and EPICOV were different. Therefore, any comparison with these two studies must be interpreted with caution. Started in March 2020, COVIPREV [40] was a study that collected weekly data on anxiety, depression (HADS scale), and sleep disorders in 2,000 adults in the French general population. Results show a prevalence of depression higher in the French general population than in our sample during the same time period (June to September 2020). Specifically, up to 11.7% of respondents in COVIPREV had depression compared to 7.6% [6.0–9.1] in our sample. The results for the prevalence of anxiety were similar in both studies (up to 17.5% in COVIPREV and 18.9% [16.5–21.2] in Aladdin) (see Fig. 5).

The modelling of the mental health risk factors in all three studies allowed us to develop and validate our multivariable model.

### International studies on health workers

Various international studies have been conducted on the mental health of hospital staff during the ongoing COVID-19 pandemic [20, 43, 44]. A prospective online cohort study in Japan [45] highlighted significantly higher levels of psychological distress and fear of COVID-19 in healthcare workers than in other workers. Some others internationals studies mentioned the consequences of lockdown on healthcare workers particularly their mental health [44, 46–48]. A cross-sectional survey in Spain [49] assessed the impact of COVID-19 on healthcare workers’ sleep quality. Results showed that sleep disorders were more frequent in the healthcare group than in the rest of the active population (*p* < 0.050) and that night-shift work was associated with a greater risk of suffering from sleep disorders.

At the beginning of the first COVID-19 wave in France, a systematic review was conducted to identify the risks factors of mental health deterioration in health workers [50]. It included findings from previous epidemics (SARS-CoV-1, H1N1) and data from the first weeks of the COVID-19 pandemic. The review described healthcare providers’ mental health vulnerability and higher risk of anxiety, depression, burnout, addiction and post-traumatic stress disorder. Consistently with our study, it identified several organizational factors as a source of anxiety, such as the lack of personal protective equipment and medical care equipment, the reassignment of one’s work position, the lack of communication, and a high level of stress at work. Familial and social difficulties (especially in terms of daily life), a lack of support from family and friends, the fear of infecting a loved one, isolation, and negative representations all impacted anxiety levels.

### Perception of night-shift work

Few studies to date have evaluated the effect of negative representations on night-workers’ mental and physical health. The consequences of negative representations are many: stress, lower self-esteem and less self-efficacy [38]. Our NSHW population suffered from two types of negative representations: perceived negative representations and self-negative representations [38]. The negative representations of night work and the stigmatization reduces night-workers’ ability to cope with the routine demands of work [51] and leads to less job satisfaction, job performance, work commitment and willingness to learn and develop [52, 53]. Underestimation, negative representations of one’s work may all be linked to a low level of social support and low personal reward and valorisation, two adverse psychosocial factors in the work environment. Moreover, psychological distress in the workplace is related to poor social support from managers and colleagues. One possible intervention to reduce the negative impact of these psychosocial factors on mental health is to provide social support at work [54, 55].

NSHW perceived their work to be particularly stigmatized and associated to negative representations [13, 14, 56, 57]. Accordingly, reducing negative representations of night work and night workers is essential in order to improve these workers’ mental health.

### Strengths and limitations

The large sample of the study allowed us to identify several dimensions of mental health of NSHW in the AP-HP network after the first wave of COVID-19 ended in France. Although NSHW are essential for the continuity of care, few data exist on this population because they are difficult to reach. One reason for this is that they have little contact with their managers and work outside of normal working hours.

A major novelty of our work is that we were able to explore the association between the negative representations of NSHW and this population’s mental health. Furthermore, our results highlight the impact of the organizational upheavals caused by the COVID-19 pandemic on mental health and sleep quality in NSHW.

An advantage of our work is that its results can have a concrete use and translation for public decision makers. Indeed, by assessing and identifying the mental health disorders of NSHW and the factors that affect them, it is possible to set up programs for prevention, surveillance and early diagnosis of these disorders.

The study also has limitations; it was only conducted with NSHW working in the public university hospitals of the AP-HP network in the region around Paris, and therefore cannot be representative of all NSHW. In addition, data concerning characteristics of NSHW who did not answer the survey questionnaire were lacking. However, the weighted analyses allowed us to ensure representativeness of the sample in terms of gender, age and professional category. Some professions such as midwifes and stretcher bearers were underrepresented as the number of these professionals who work at night is very low in France. Finally, some data were not collected and could have been used to refine the results, such as the number of hours of sleep.

## Conclusion and perspectives

Our study shows high prevalences although lower than in the general population of depression, anxiety, sleep disorders, and symptoms suggestive of post-traumatic stress disorder among NSHW, a category of healthcare providers already facing specific health challenges due to perturbations of circadian rhythms. This confirms the importance of monitoring mental health and sleep quality in this population, even more during periods of health crises such as the COVID-19 pandemic. Findings also highlight the deleterious effect of both negative representations of night work and changes in work organization during the pandemic on all mental health outcomes. nMoreover, multilevel interventions aiming at reducing negative representations and improving work organization in NSHW are urgently needed to improve the overall health in this key healthcare providers group.

## Supplementary Information

**Additional file 1:** **Appendix 1. **Main characteristics of night shift healthcare workers according to profession(*n*=1200, ALADDIN survey. **Appendix 2**. Complete and multinomial regressions of factors associated with probable and definite anxiety, probable and depression, moderate and severe insomnia and acute and post-traumatic stress.

## Data Availability

The datasets generated and analyzed during the current study are not publicly available due to the French legislation on research involving the human person and the presence of potentially identifying or sensitive patient information. The datasets used and analyzed are available from the corresponding author upon reasonable request.

## Abbreviations

NSHW: Night-shift healthcare workers
HADS: Hospital Anxiety and Depression Scale
AP-HP: Public hospitals in the Paris area
ISI: Insomnia severity index
IES-R: Impact of event scale—revised
aRRR: Adjusted relative risk ratios
