# Supplementary material for: Negative representations of night-shift work and mental health of public hospital healthcare workers in the COVID-19 era (Aladdin survey)
Source: BMC Health Serv Res. 2023 Feb 22;23:187. doi: 10.1186/s12913-023-09101-7 (PMC9946706; doi:10.1186/s12913-023-09101-7)
Supplement: Supplementary file 1 — Additional file 1: Appendix 1. Main characteristics of night shift healthcare workers according to profession(n=1200, ALADDIN survey. Appendix 2. Complete and multinomial regressions of factors associated with probable and definite anxiety, probable and depression, moderate and severe insomnia and acute and post-traumatic stress. [file 12913_2023_9101_MOESM1_ESM.docx]

**APPENDIX 1: Main characteristics of night shift healthcare workers according to profession(n=1200, ALADDIN survey)**

HCW = healthcare workers; IQR = interquartile range; SD = standard deviation.

^1^ Comparison of characteristics between the five professional categories of HCW (Chi-square tests for categorical variables, Wald test for continuous variables).

|  |  | **Professional category of HCW** | | | | |  |
| --- | --- | --- | --- | --- | --- | --- | --- |
| **Characteristics** | **Whole study population** | **Nurses** | **Assistant nurses/ technicians** | **Midwives** | **Managers** | **Other** | ***p-value^1^*** |
|  | (n=1,200) | (53.6%) | (36.9%) | (4.1%) | (0.8%) | (4.6%) |  |
|  | **% of HCW, mean (SD) or median [IQR]** | | | | | |  |
| **Socio-demographic and economic characteristics** | | | | | | | |
| **Female gender** | 78.2 [75.7-80.8] | 82.4 | 73.3 | 95.4 | 69.8 | 55.9 | ***<0.001*** |
| **Age** *- in years* | 39.4 (11.8) | 36.3 (10.5) | 44.7 (12.1) | 32.2 (8.1) | 53.6 (4.3) | 38.8 (19.0) | ***<0.001*** |
| **Matrimonial status** |  |  |  |  |  |  | ***<0.001*** |
| - single | 36.2 [33.4-39.1] | 42.3 | 28.4 | 31.1 | 14.7 | 38.0 |  |
| - in cohabitation | 20.6 [18.2-23] | 20.8 | 18.7 | 36.5 | 15.2 | 20.6 |  |
| - PACS or married | 33.7 [30.9-36.4] | 30.0 | 40.3 | 32.4 | 46.5 | 21.9 |  |
| - widow/widower | 9.5 [7.7-11.3] | 7.0 | 12.6 | 0 | 23.6 | 19.5 |  |
| **Has children** |  |  |  |  |  |  | ***<0.001*** |
| - no | 49.3 [46.4-52.3] | 60.0 | 31.3 | 71.7 | 26.4 | 54.2 |  |
| - yes (mean numbers of children) | 50.7 [47.7-53.6] | 40.0 | 68.8 | 28.3 | 73.6 | 45.8 |  |
| **Perceived financial status** |  |  |  |  |  |  | ***<0.001*** |
| - Financially comfortable/ gets by | 40.3 [37.4-43.2] | 45.8 | 28.1 | 85.3 | 63.8 | 29.9 |  |
| - Has to be careful | 46.4 [43.5-49.3] | 46.2 | 50.9 | 14.7 | 33.3 | 42.8 |  |
| - Financial difficulties | 13.3 [11.2-15.5] | 8.0 | 21.0 | 0 | 3.0 | 27.3 |  |
| **Mental health characteristics** |  |  |  |  |  |  |  |
| **HADS score** |  |  |  |  |  |  |  |
| **Anxiety** |  |  |  |  |  |  | ***0.011*** |
| -          Normal | 60.5 [57.6-63.4] | 59.2 | 64.4 | 68.1 | 63.4 | 37.5 |  |
| -          Doubtful presence | 20.6 [18.2-23.0] | 21.4 | 19.5 | 15.4 | 12.7 | 26.2 |  |
| -          Caseness | 18.9 [16.5-21.2] | 19.4 | 16.1 | 16.5 | 23.9 | 36.4 |  |
| **Depression** |  |  |  |  |  |  |  |
| -          Normal | 78.5 [76.1-81.0] | 77.9 | 80.3 | 91.2 | 77.1 | 60.0 |  |
| -          Doubtful presence | 13.9 [11.8-16.0] | 14.7 | 11.8 | 4.1 | 15.3 | 30.1 |  |
| -          Caseness | 7.6 [6.0-9.1] | 7.4 | 8.0 | 4.7 | 7.7 | 9.9 |  |
| **Insomnia Severity Index** |  |  |  |  |  |  | ***0.010*** |
| -          Absence of insomnia | 24.0 [21.4-26.5] | 19.8 | 27.8 | 34.4 | 30.1 | 30.6 |  |
| -          Sub clinical insomnia (light) | 34.4 [31.6-37.2] | 35.4 | 32.5 | 34.5 | 38.1 | 35.4 |  |
| -          Clinical insomnia (moderate) | 33.1 [30.4-35.9] | 35.2 | 31.9 | 31.1 | 26.3 | 22.0 |  |
| -          Clinical insomnia (severe) | 8.6 [6.9-10.2] | 9.5 | 7.7 | 0 | 5.6 | 12.0 |  |
| **Post-traumatic Stress** |  |  |  |  |  |  | *0.057* |
| -          No particular stress | 72.5 [69.8-75.3] | 74.3 | 69.5 | 88.1 | 73.7 | 60.9 |  |
| -          Acute stress | 15.8 [13.5-18.1] | 15.0 | 17.8 | 7.3 | 10 | 19.0 |  |
| -          Post-traumatic stress | 11.7 [9.7-13.6] | 10.7 | 12.8 | 4.7 | 16.3 | 20.1 |  |
| **Work-related characteristics** |  |  |  |  |  |  |  |
| **Type of position** |  |  |  |  |  |  | ***<0.001*** |
| - Night permanent position | 76.2 [73.6-78.8] | 76.9 | 85.0 | 0 | 85.7 | 63.1 |  |
| - Replacement | 4.6 [3.4-5.8] | 4.7 | 5.0 | 0 | 0 | 4.3 |  |
| - Position with day/night alternation | 16.1 [13.8-18.4] | 15.9 | 8.1 | 100 | 4.3 | 10.4 |  |
| - New night position during the COVID 19 epidemic | 0.5 [0.1-0.9] | 0.8 | 0.3 | 0 | 0 | 0 |  |
| - Other | 2.6 [1.4-3.8] | 1.7 | 1.6 | 0 | 10.1 | 22.2 |  |
| **Hospital department** |  |  |  |  |  |  | ***<0.001*** |
| - Pediatric | 15.2 [13.1-17.3] | 17.3 | 15.6 | 0 | 1.6 | 4.1 |  |
| - Adults | 61.0 [58.1-63.9] | 65.9 | 60.7 | 41.7 | 23.5 | 29.8 |  |
| - Several services | 23.8 [21.2-26.4] | 16.8 | 23.8 | 58.3 | 74.9 | 66.1 |  |
| **Seniority as a night-shift worker** *- in years* | 9.2 (8.5) | 8.4 (7.7) | 10.4 (9.8) | 8.4 (8.2) | 15.5 (4.8) | 7.6 (13.7) | ***<0.001*** |
| **Daily duration of work** |  |  |  |  |  |  | ***<0.001*** |
| - 10 hours | 62.7 [59.8-65.6] | 61.9 | 72.6 | 0 | 83.3 | 44.1 |  |
| - 12 hours | 33.6 [30.9-36.4] | 35.9 | 24.9 | 95.9 | 7.0 | 27.0 |  |
| - other | 3.7 [2.4-5.0] | 2.2 | 2.5 | 4.1 | 9.7 | 29.0 |  |
| **Part-time work** |  |  |  |  |  |  | ***0.118*** |
| -          Yes | 4.8 [3.6-6.1] | 5.5 | 3.2 | 10.9 | 8.2 | 3.7 |  |
| -          No | 95.2 [93.9-96.4] | 94.5 | 96.80 | 89.1 | 91.8 | 96.3 |  |
| **Travel time to work** - *in hours* | 0.7 (0.6) | 0.7 (0.5) | 0.8 (0.7) | 0.7 (0.4) | 0.6 (0.2) | 0.9 (0.6) | *0.066* |
| **Health-related characteristics** |  |  |  |  |  |  |  |
| **Physical activity** |  |  |  |  |  |  | ***<0.001*** |
| -          No | 45.8 [42.8-48.7] | 47.5 | 46.3 | 14.5 | 60.8 | 47.2 |  |
| -          Yes | 54.2 [51.3-57.2] | 52.5 | 53.7 | 85.5 | 39.2 | 52.8 |  |
| **History of psychiatric troubles (depression, bipolar disorders, etc.)** |  |  |  |  |  |  | ***<0.001*** |
| -          No | 94.9 [93.5-96.2] | 95.9 | 95.4 | 92.2 | 89.1 | 82.4 |  |
| -          Yes | 5.1 [3.8-6.5] | 4.1 | 4.6 | 7.8 | 10.9 | 17.6 |  |
| **History of harassment at work** |  |  |  |  |  |  | *0.320* |
| -          No | 79.0 [76.6-81.5] | 79.3 | 78.2 | 88.0 | 60.4 | 76.7 |  |
| -          Yes | 21.0 [18.5-23.5] | 20.7 | 21.8 | 12.1 | 39.6 | 23.3 |  |
| **History of SARS-CoV-2 infection** |  |  |  |  |  |  | *0.860* |
| -          No | 81.5 [78.9-84.2] | 80.4 | 83.0 | 79.7 | 82.2 | 85.5 |  |
| -          Yes | 18.5 [15.8-21.1] | 19.7 | 17.0 | 20.3 | 17.8 | 14.5 |  |
| **Alcohol consumption** |  |  |  |  |  |  | ***<0.001*** |
| -          Never | 30.6 [27.8-33.4] | 25.4 | 39.1 | 27.5 | 35.3 | 25.3 |  |
| -          Monthly | 47.7 [44.8-50.7] | 49.7 | 47.4 | 18.8 | 49.8 | 52.2 |  |
| -          Weekly | 20.3 [17.9-22.7] | 23.6 | 13.0 | 42.8 | 9.3 | 22.6 |  |
| -          Everydays | 1.4 [0.7-2.1] | 1.2 | 0.6 | 10.9 | 5.7 | 0 |  |
| **Screen consumption** |  |  |  |  |  |  | ***<0.001*** |
| -          IAS score med (Q1;Q3) | 2.3 (2.0) | 2.5 (1.8) | 2.0 (2.2) | 3.1 (3.1) | 1.5 (0.8) | 2.3 (4.0) |  |
| **WORK-related Perceptions** |  |  |  |  |  |  |  |
| **Night-shift work is under-estimated by colleagues working on day** |  |  |  |  |  |  | ***<0.001*** |
| -          Always, regularly | 65.0 [62.1-67.8] | 67.1 | 70.5 | 26.9 | 74.4 | 28.2 |  |
| **Night-shift work is under-estimated by loved ones** |  |  |  |  |  |  | ***0.024*** |
| -          Always, regularly | 21.0 [18.6-23.4] | 23.1 | 17.5 | 33.5 | 13.3 | 14.7 |  |
| **Night-shift work is under-estimated by patients** |  |  |  |  |  |  | *0.582* |
| -          Always, regularly | 17.7 [15.5-19.9] | 19.4 | 16.0 | 14.7 | 16.2 | 14.7 |  |
| **Day duties are more important than night duties in term of patient care** |  |  |  |  |  |  | *0.610* |
| -          strongly agree, agree | 23.5 [21.0-26.0] | 23.2 | 24.8 | 15.3 | 16.5 | 25.6 |  |
| **Day workload is higher than night workload** |  |  |  |  |  |  | ***<0.001*** |
| -          strongly agree, agree | 39.1 [36.2-41.9] | 43.3 | 33.6 | 22.4 | 17.2 | 52.7 |  |
| **Feels more irritable since works at night** |  |  |  |  |  |  | ***<0.001*** |
| -          strongly agree, agree | 43.7 [40.8-46.7] | 48.8 | 33.8 | 72.8 | 30.1 | 40.3 |  |
| **Work organization: changes since the beginning of the COVID-19 pandemic** | | | | | | | |
| **No change at all** | 37.4 [34.5-40.3] | 31.9 | 46.0 | 26.6 | 24.7 | 45.3 | ***<0.001*** |
| **Change of department** | 25.1 [22.6-27.6] | 28.0 | 24.6 | 3.4 | 16.1 | 16.2 | ***0.001*** |
| **Increase of the no. of working hours** | 36.8 [33.9-39.6] | 39.4 | 27.1 | 70.1 | 62.6 | 49.1 | ***<0.001*** |
| **Switch to nightshift work** | 4.8 [3.5-6.1] | 5.1 | 3.4 | 0 | 4.9 | 18.4 | ***<0.001*** |
| **Change of activity to manage COVID patients** | 18.6 [16.4-20.8] | 24.1 | 13.4 | 11.4 | 9.1 | 4.5 | ***<0.001*** |
| **Feeling related to the COVID-19 pandemic** | | | | | | | |
| **Satisfied of the information on COVID received from the employer** |  |  |  |  |  |  | *0.353* |
| -   No information received | 10.0 [8.2-11.8] | 9.7 | 11.2 | 3.4 | 3.9 | 10.0 |  |
| -   Are insufficient | 58.5 [55.6-61.4] | 60.0 | 56.9 | 66.5 | 40.1 | 50.0 |  |
| -    Are sufficient | 31.5 [28.8-34.3] | 30.3 | 31.9 | 30.1 | 56.0 | 40.0 |  |
| **Feels vulnerable to COVID-19 because of professional activity** | 77.7 [75.2-80.2] | 80.3 | 73.5 | 89.4 | 63.0 | 72.9 | ***0.014*** |
| **Fears to get the COVID-19 at work** | 65.6 [62.8-68.5] | 64.2 | 67.4 | 73.7 | 44.3 | 65.0 | *0.337* |
| **Fears to transmit the COVID-19 to close relatives** | 90.8 [89.1-92.6] | 90.4 | 91.0 | 96.5 | 79.3 | 91.3 | *0.464* |
| **Has received psychological support from close relatives during the pandemic** | 7.0 [5.4-8.6] | 7.6 | 7.4 | 0 | 2.0 | 4.3 | *0.284* |
| **Has received psychological support from a professional during the pandemic** | 8.3 [6.6-9.9] | 8.4 | 9.1 | 0 | 3.4 | 8.1 | *0.277* |
| **Felt valued by the general population as a HCW during the pandemic** |  |  |  |  |  |  | *0.077* |
| -          strongly agree, agree | 63.1 [60.2-66.0] | 65.0 | 59.6 | 76.9 | 63.0 | 55.8 |  |
| **Is confident in the health authorities to manage the crisis** |  |  |  |  |  |  | *0.344* |
| -          strongly agree, agree | 19.7 [17.3-22.1] | 18.7 | 20.5 | 23.1 | 43.1 | 17.7 |  |
| **Faced difficulties in applying protective measures against COVID** |  |  |  |  |  |  | *0.468* |
| -          strongly agree, agree | 59.9 [56.9-62.8] | 59.3 | 59.2 | 69.1 | 44.9 | 65.9 |  |
| **Considers protectives measures inadequate** |  |  |  |  |  |  | *0.750* |
| -          strongly agree, agree | 27.4 [24.8-30.1] | 27.7 | 28.1 | 23.6 | 10.9 | 25.7 |  |
| **Faced difficulties in getting screened for SARS-CoV-2 infection** |  |  |  |  |  |  | ***0.014*** |
| -          strongly agree, agree | 58.3 [55.4-61.3] | 57.8 | 62.1 | 56.9 | 37.3 | 38.8 |  |

**APPENDIX 2: Complete and multinomial regressions of factors associated with probable and definite anxiety, probable and depression, moderate and severe insomnia and acute and post-traumatic stress**

|  | **HAD Anxiety**  **(ref: Absence of symtpomatology)** | | **HAD depression**  **( ref: Absence of symptomatology)** | | **Index of insomnia severity**  **( ref: Absence or Sub-threshold )** | | | **Post-traumatic stress**  **( ref: No particular stress )** | |
| --- | --- | --- | --- | --- | --- | --- | --- | --- | --- |
|  | **Doubtful presence** | **Definite case** | **Doubtful presence** | **Definite case** | **Moderate insomnia** | **Severe insomnia** | | **Acute stress** | **Post-traumatic stress** |
| **Characteristics** | **aRRR^1^ [95% CI]** | | **aRRR^1^ [95% CI]** | | **aRRR^1^ [95% CI]** | | **aRRR^1^ [95% CI]** | | |
| **Female gender ^$^** | 1.15 [0.78-1.70] | **1.98 [1.26-3.11]** |  |  |  |  | |  |  |
| **Age** *- in years* | 1.00 [0.98-1.02] | **0.97 [0.95-1.00]** |  |  | 0.99 [0.98-1.00] | **0.94 [0.92-0.97]** | |  |  |
| **Perceived financial status** |  |  |  |  |  |  | |  |  |
| - Feels financially comfortable/it’s okay | ref | ref | ref | ref | ref | ref | | ref | ref |
| - Has to be careful | 1.33 [0.93-1.89] | **1.65 [1.12-2.42]** | **1.96 [1.30-2.96]** | **1.83 [1.05-3.20]** | 1.32 [0.99-1.77] | **2.20 [1.29-3.77]** | | **2.72 [1.85-4.01]** | **2.48 [1.54-3.99]** |
| - Faces financial difficulties | **2.10 [1.24-3.57]** | **3.46 [2.00-5.98]** | **3.30 [1.90-5.75]** | **3.15 [1.52-6.54]** | 1.34 [0.86-2.10] | **4.68 [2.31-9.47]** | | **2.33 [1.32-4.10]** | **4.33 [2.38-7.85]** |
| **Work-related characteristics** |  |  |  |  |  |  | |  |  |
| **Professional category** |  |  |  |  |  |  | |  |  |
| - Nurses | ref | ref | ref | ref |  |  | |  |  |
| - Assistant nurses/technicians | 0.90 [0.62-1.30] | 0.85 [0.57-1.26] | **0.61 [0.41-0.91]** | 0.78 [0.47-1.29] |  |  | |  |  |
| - Midwives | 0.46 [0.18-1.18] | 0.45 [0.17-1.16] | 0.40 [0.09-1.72] | 1.38 [0.33-5.83] |  |  | |  |  |
| - Managers | 1.00 [0.13-7.57] | 2.54 [0.42-15.49] | 1.19 [0.20-7.23] | 0.73 [0.04-13.53] |  |  | |  |  |
| - Other | **2.86 [1.28-6.39]** | **2.51 [1.07-5.87]** | **2.51 [1.24-5.09]** | 0.83 [0.20-3.47] |  |  | |  |  |
| **Type of position** |  |  |  |  |  |  | |  |  |
| - Night permanent position | ref | ref |  |  | ref | ref | |  |  |
| - Replacement (’’pool’’) | **0.18 [0.05-0.63]** | 0.46 [0.18-1.18] |  |  | **0.29 [0.12-0.69]** | 0.44 [0.12-1.67] | |  |  |
| - Position with day/night alternation | **1.95 [1.16-3.28]** | 1.70 [0.99-2.92] |  |  | **1.56 [1.05-2.32]** | 1.63 [0.84-3.16] | |  |  |
| - New night position during the COVID 19 epidemic | 0.31 [0.03-3.64] | 1.39 [0.23-8.59] |  |  | 7.29 [0.65-81.68] | 1.81 [0.07-45.03] | |  |  |
| - Other | 0.41 [0.10-1.68] | 0.80 [0.23-2.81] |  |  | 0.51 [0.17-1.55] | 1.60 [0.47-5.48] | |  |  |
| **Seniority as a night-shift worker** *- in years* | **0.97 [0.94-1.00]** | 1.02 [0.99-1.05] |  |  |  |  | | 0.99 [0.97-1.01] | **0.96 [0.94-0.99]** |
| **Part-time work ^#^** |  |  |  |  | **0.68 [0.50-0.93]** | 0.79 [0.46-1.34] | |  |  |
| **Health-related characteristics** |  |  |  |  |  |  | |  |  |
| **Physical activity ^#^** |  |  | **0.52 [0.37-0.75]** | **0.55 [0.34-0.89]** | **0.69 [0.53-0.90]** | **0.62 [0.39-0.98]** | |  |  |
| **History of psychiatric troubles (depression, bipolar disorders, etc.) ^#^** | **2.31 [1.10-4.85]** | **4.19 [2.10-8.35]** | **2.07 [1.08-3.97]** | 1.57 [0.60-4.10] | **1.85 [1.01-3.36]** | **2.54 [1.06-6.08]** | |  |  |
| **History of harassment at work ^#^** | 1.06 [0.71-1.58] | **1.62 [1.10-2.40]** |  |  | **1.42 [1.02-1.97]** | **1.82 [1.06-3.12]** | | 1.22 [0.81-1.84] | **2.85 [1.87-4.36]** |
| **History of SARS-CoV-2 infection** |  |  |  |  |  |  | |  |  |
| -          No | ref | ref | ref | ref | ref | ref | |  |  |
| -          Yes | **1.81 [1.15-2.84]** | 1.43 [0.86-2.39] | **1.84 [1.15-2.97]** | 1.09 [0.55-2.18] | 1.20 [0.80-1.80] | **2.02 [1.10-3.69]** | |  |  |
| - Doesn’t know | 1.09 [0.75-1.58] | 1.39 [0.96-2.03] | 0.91 [0.59-1.38] | 0.94 [0.55-1.62] | 1.22 [0.90-1.65] | 0.77 [0.44-1.34] | |  |  |
| **Work-related perceptions** |  |  |  |  |  |  | |  |  |
| **Night-shift work is under-estimated by colleagues working on day ^¤^** |  |  |  |  |  |  | |  |  |
| -          Always, regularly | **1.75 [1.19-2.59]** | 0.84 [0.57-1.24] | 1.01 [0.68-1.50] | **2.10 [1.16-3.82]** | **1.53 [1.12-2.08]** | 1.48 [0.86-2.57] | |  |  |
| **Night-shift work is under-estimated by patients ^¤^** |  |  |  |  |  |  | |  |  |
| -          Always, regularly | **1.54 [1.04-2.28]** | **1.86 [1.23-2.79]** | 1.15 [0.74-1.79] | **2.02 [1.21-3.39]** | **1.64 [1.17-2.30]** | **1.81 [1.05-3.12]** | |  |  |
| **Day duties are more important than night duties in terms of patient care^*^** |  |  |  |  |  |  | |  |  |
| -          Strongly agree, agree | 1.16 [0.80-1.70] | **1.50 [1.02-2.20]** | **1.70 [1.14-2.52]** | **2.25 [1.36-3.74]** | **1.46 [1.06-2.00]** | **2.27 [1.38-3.75]** | | 1.16 [0.77-1.75] | **1.96 [1.26-3.04]** |
| **Work organization: changes since the beginning of the COVID-19 pandemic** |  |  |  |  |  |  | |  |  |
| **Switch to nightshift work #** |  |  | 0.89 [0.36-2.18] | **3.22 [1.37-7.57]** |  |  | |  |  |
| **Change of activity to manage COVID patients ^#^** | 1.02 [0.67-1.54] | **1.54 [1.03-2.31]** |  |  | 1.13 [0.80-1.59] | **1.81 [1.07-3.05]** | | **1.57 [1.04-2.36]** | **2.07 [1.30-3.28]** |
| **Feelings related to the COVID-19 pandemics** |  |  |  |  |  |  | |  |  |
| **Satisfied of the information on COVID received from the employer** ^#^ | 0.90 [0.64-1.28] | **0.48 [0.32-0.73]** |  |  |  |  | | **0.54 [0.35-0.82]** | 0.94 [0.59-1.48] |
| **Fears to get the COVID-19 at work ^#^** | 1.06 [0.76-1.47] | 1.75 [1.19-2.56] | **3.42 [1.38-8.45]** | 1.13 [0.49-2.59] |  |  | | **1.83 [1.24-2.71]** | **2.12 [1.31-3.44]** |
| **Fears to transmit the COVID-19 to close relatives ^#^** |  |  |  |  | **1.70 [1.00-2.87]** | 1.38 [0.52-3.67] | |  |  |
| **Faced difficulties in applying protective measures against COVID ^*^** |  |  |  |  |  |  | |  |  |
| -          strongly agree, agree | **1.92 [1.37-2.70]** | 1.24 [0.88-1.76] |  |  |  |  | |  |  |
| **Considers protectives measures inadequate ^*^** |  |  |  |  |  |  | |  |  |
| -          strongly agree, agree |  |  |  |  | 1.19 [0.89-1.60] | **1.90 [1.18-3.06]** | | 1.13 [0.77-1.64] | **1.62 [1.06-2.48]** |

**^1^** aRRR = adjusted Relative Risk Ratio; aRR [95% CI] in **bold** means that pvalue associated is lower than 0.05

**^$^** Reponse “Male” set as reference

^#^ Response “No” set as reference (modality Yes is tested)

**^*^** Responses “strongly disagree”, “disagree”or “indifferent” were gathered and set as reference

**^¤^** Responses “Never”, “rarely”, or “sometimes” were gathered and set as reference
